# Dynamische Preisgestaltung in der digitalisierten Welt

**DOI:** 10.1007/s41471-020-00095-0

**Published:** 2020-06-26

**Authors:** Martin Spann, Bernd Skiera

**Affiliations:** 1 Institut für Electronic Commerce und Digitale Märkte, Ludwig-Maximilians-Universität München, Ludwigstr. 28, 80539 München, Deutschland; 2 Professur für Betriebswirtschaftslehre, insbesondere Electronic Commerce, Goethe-Universität Frankfurt am Main, Theodor-W.-Adorno-Platz 4, 60323 Frankfurt am Main, Deutschland

**Keywords:** Dynamic Pricing, Preisdifferenzierung, Preisdiskriminierung, Digitalisierung, Privatsphäre, Datenschutz, Dynamic pricing, Price differentiation, Price discrimination, Digitization, Privacy, M30, D40, D10

## Abstract

Digitale Technologien begünstigen den Einsatz einer dynamischen Preisgestaltung, also von Preisen, die für ein prinzipiell gleiches Produkt unangekündigt variieren. Dabei werden in der öffentlichen Diskussion unterschiedliche Ausgestaltungsformen dynamischer Preise oftmals vermischt, was eine sinnvolle Analyse der Vor- und Nachteile der dynamischen Preisgestaltung erschwert. Das Ziel des Beitrags ist die Darstellung der ökonomischen Grundlagen und die Diskussion sowie Klassifikation der Ausgestaltungsmöglichkeiten der dynamischen Preisgestaltung. Darüber hinaus erfolgt eine Bewertung der Vor- und Nachteile der dynamischen Preisgestaltung aus Käufer- und Verkäufersicht. Abschließend werden Implikationen für die betriebswirtschaftliche Forschung diskutiert.

## Abstract (en)

Digital technologies favor the use of dynamic pricing, i.e., prices that vary unannounced for a product that basically remains unchanged. However, different forms of dynamic pricing are often mixed in the public discussion, which makes a meaningful analysis of the advantages and disadvantages of dynamic pricing difficult. The aim of this paper is to present the economic foundations of dynamic pricing as well as to discuss and to classify its design options. In addition, the paper assesses dynamic pricing from a buyer and seller perspective. Finally, the paper discusses implications for business research.

## Problemstellung

Dynamische Preisgestaltung bezeichnet eine Preisstrategie, bei der die Preise für ein prinzipiell gleiches Produkt, hier definiert als ein Gut oder eine Dienstleistung, über Kaufzeitpunkte oder Konsumenten variieren. Diese breite Definition der dynamischen Preisgestaltung beinhaltet sowohl, dass diese Anpassung im Zeitverlauf (beispielsweise als Reaktion auf eine Nachfrageänderung) erfolgen kann, als auch, dass zu einem Kaufzeitpunkt unterschiedliche Preise von verschiedenen Käufern verlangt werden.^1^ In der Regel werden bei dynamischer Preisgestaltung die Preise häufiger, u. U. sogar mehrmals täglich, angepasst als im Vergleich zu Situationen ohne dynamische Preisgestaltung.

Das wesentliche Unterscheidungsmerkmal dynamischer Preisgestaltung zu anderen Formen der Preisdifferenzierung besteht darin, dass die Preisvariation im Vorhinein nicht angekündigt wird. Beispielsweise ist eine zeitliche Variation der Preise bei Kinofilmen angekündigt, wohingegen die verschiedenen Preise eines Fluges (u. a. in Abhängigkeit vom Buchungszeitpunkt) nicht angekündigt werden. Ebenso können unterschiedliche Preise für verschiedene Personen oder Gruppen angekündigt sein (z. B. Studierendentarif und Normaltarif) oder nicht (z. B. unterschiedliche Preise für eine Person, z. B. nach Zugriffsgerät (Android vs. iOS) oder Zugriffkanal (direkter Einstieg auf die Webseite oder Preisvergleichsseite) auf den Online-Shop). Auf Basis dieser Definition bilden „Yield Management“ oder „Revenue Management“-Anwendungen, deren Fokus im Bereich Luftverkehr auf die optimale Auslastung der Sitzplätze in Flugzeugen ausgerichtet ist (Talluri und van Ryzin 2004), einen Teilbereich der dynamischen Preisgestaltung.

Vor Mitte der 1990er Jahre war das Spektrum möglicher Anwendungen dynamischer Preisgestaltung dadurch begrenzt, dass die Kosten für die Kommunikation variierender Preise an potenzielle Käufer und die Erfassung von Käufen mit variierenden Preise hoch waren. Im Bereich Luftverkehr erfolgten Preisänderungen über zentrale Buchungssysteme, die Fluglinien mit Reisebüros verbanden (und dies noch heute tun). Das Internet als interaktives Medium bietet die genannten Voraussetzungen einer aktuellen Preiskommunikation und -reaktion (Kauf bzw. Buchung) und hat daher schon seit Mitte der 1990er Jahren Endkunden in direkten Kontakt mit dynamischer Preisgestaltung gebracht (Spann et al. 2005).

Die Erfahrungen aus der Luftverkehrsbranche, aber auch die stark variierenden Preise an Tankstellen zeigen, dass Kunden dynamische Preise akzeptieren. In den letzten Jahren hat sich die Anwendung dynamischer Preise, begünstigt durch die Verfügbarkeit erhöhter Datenmengen und leistungsfähigerer IT-Infrastruktur, insbesondere auf den Bereich des Online-Handels ausgeweitet (Chen et al. 2016). Das hat dazu geführt, dass die dynamische Preisgestaltung auch vermehrtes Interesse in der öffentlichen Wahrnehmung erfährt und dort oftmals kritisch betrachtet wird.^2^

Dabei werden in der öffentlichen Diskussion unterschiedliche Formen dynamischer Preise oftmals vermischt, was die Analyse der Vor- und Nachteile sowie der Einflussfaktoren auf diese Preisgestaltung erschwert. Das Ziel des Beitrags ist es, zu einer sachlicheren Diskussion durch die Darstellung der ökonomischen Grundlagen und die Diskussion sowie Klassifikation der Ausgestaltungsmöglichkeiten der dynamischen Preisgestaltung beizutragen. Darüber hinaus erfolgen eine Beurteilung der dynamischen Preisgestaltung aus Käufer- und Verkäufersicht und eine Diskussion des weiteren Forschungsbedarfs.

Nachfolgend stellt Abschn. 2 die ökonomischen Grundlangen der Preisdifferenzierung dar und ordnet die dynamische Preisgestaltung in die Formen der Preisdifferenzierung ein. Abschn. 3 klassifiziert die unterschiedlichen Formen dynamischer Preisgestaltung. Darauf aufbauend diskutiert Abschn. 4 die Ausgestaltung dynamischer Preise und unterscheidet zwischen ökonomischen und verhaltenswissenschaftlichen Einflussfaktoren sowie Datenanforderungen und rechtlichen Überlegungen. Abschn. 5 diskutiert dynamische Preisgestaltung aus Verkäufer- und Käufersicht. Der Beitrag schließt in Abschn. 6 mit einer Diskussion der Implikationen und des weiteren Forschungsbedarfs zur dynamischen Preisgestaltung.

## Preisdifferenzierung

### Ökonomische Grundlagen der Preisdifferenzierung

Die ökonomische Grundlage der Preisdifferenzierung besteht in der Heterogenität von Käuferbedürfnissen und damit einhergehenden unterschiedlichen Zahlungsbereitschaften. Der fallende Verlauf von Preisabsatzfunktionen reflektiert die Heterogenität der Zahlungsbereitschaften: mit steigenden Preis gibt es weniger Käufer im Markt, weil deren Zahlungsbereitschaft gleich oder über dem Marktpreis liegen muss.^3^

**Figure 1:**
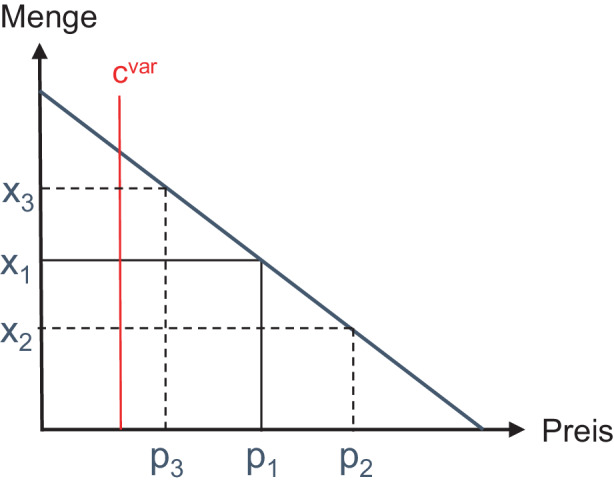

In Abb. 1 stellt der Preis p_1_ eine Situation dar, bei der x_1_ Käufer (falls nur 1 Einheit je Käufer erworben wird) kaufen würden. Allerdings entgeht dem Anbieter in der Kombination (p_1_, x_1_) Deckungsbeitrag, da i) bis auf den marginalen Käufer (x_1_) alle Käufer eine höhere Zahlungsbereitschaft gehabt hätten (und somit auch höhere Preise akzeptiert hätten) und ii) Käufer, deren Zahlungsbereitschaft über den variablen Kosten c^var^, aber unter dem aktuellen Preis liegt, nicht gekauft haben, obwohl dies zusätzlichen Deckungsbeitrag ermöglicht hätte (Olderog und Skiera 2000). Mit den zusätzlichen Preisen p_2_ und p_3_ kann daher weiterer Deckungsbeitrag erwirtschaftet werden.

### Formen der Preisdifferenzierung

Die Möglichkeiten zur Preisdifferenzierung unterscheiden sich im Wesentlichen darin, ob ein Anbieter entweder Käufer in unterschiedliche Gruppen aufteilt und jeder dieser Gruppen einen eigenen Preis anbietet oder ob der Anbieter das prinzipiell gleiche Produkt in verschiedenen Varianten zu unterschiedlichen Preisen anbietet und sich die Käufer selbst die für sie geeignete Variante mit dem damit verbundenen Preis heraussuchen können. Der erste Fall wird als Preisdifferenzierung „ohne Selbstselektion“ und der zweite Fall als Preisdifferenzierung „mit Selbstselektion“ bezeichnet (Skiera 1999, S. 140).

#### Preisdifferenzierung ohne Selbstselektion

Im Extremfall einer Preisdifferenzierung ohne Selbstselektion erhält jeder potenzielle Käufer seinen individuellen Preis. Im Idealfall für den Verkäufer entspricht jeder Preis genau der Zahlungsbereitschaft des Käufers (sofern diese über den variablen Kosten des Produkts liegt). Dieser Idealfall wird auch als Preisdifferenzierung ersten Grades bezeichnet (Pigou 1929). Eine derartige Preisgestaltung erfordert im idealtypischen Zustand die Kenntnis der individuellen Zahlungsbereitschaften aller potenziellen Käufer. Allerdings stellt dies hohe Anforderungen an die Datenqualität, zumal die Käufer bei einer derartigen Preisgestaltung natürlich kein Interesse am Aufdecken ihrer Zahlungsbereitschaft haben. Zudem darf aus juristischer Sicht keine Diskriminierung (z. B. auf Basis von Geschlecht) vorliegen (an der Heiden und Wersig 2018, S. 130 ff.). Darüber hinaus muss eine solche Preisdifferenzierung auch gesellschaftlich akzeptiert sein. Gesellschaftlich akzeptiert ist beispielsweise, dass Familien, Studierende oder sozial Benachteiligte günstigere Preise erhalten.

Sofern potenzielle Käufer anhand von Gruppencharakteristika in verschiedene Preisgruppen eingeteilt werden, liegt eine gruppenbezogene Preisdifferenzierung ohne Selbstselektion vor, die als Preisdifferenzierung dritten Grades bezeichnet wird (Pigou 1929). Dabei kann die Einteilung in Gruppen anhand geographischer Merkmale (Standort des Käufers) oder Merkmalen der Käufer (z. B. Studierendentarife) erfolgen.

#### Preisdifferenzierung mit Selbstselektion

Bei der Preisdifferenzierung mit Selbstselektion werden unterschiedliche Varianten des prinzipiell gleichen Produkts angeboten und potenziellen Käufern die Auswahl der für sie besten Produktvariante zu dem damit verbundenen Preis überlassen. Ein typisches Beispiel ist Bahnfahren in der ersten und zweiten Klasse. Das Produkt ist prinzipiell gleich, weil jeder Reisende der Bahn die gleiche Strecke transportiert wird. Exakt gleich kann es aber nicht sein, weil ja sonst jeder Reisende die günstigste Variante wählen würde. Die Produktvarianten unterscheiden sich also beispielsweise im Sitzkomfort oder der Ruhe im Abteil, so dass Reisende der ersten Klasse auch einen höheren Preis zu zahlen bereit sind.

Die Herausforderung für Anbieter einer Preisdifferenzierung mit Selbstselektion besteht also darin, die Produkte anhand mindestens einer Dimension unterschiedlich zu gestalten, damit potenzielle Käufer auch andere als nur die günstigste Variante wählen. Gängige Dimensionen zur differenzierten Gestaltung von Produkten sind a) Zeitpunkt des Kaufs oder der Nutzung, b) Produktqualität, c) Quantität des Produkts und d) erforderlicher Aufwand zur Suche der günstigeren Preisvarianten (Skiera und Spann 2000).

In der Regel wird Preisdifferenzierung mit Selbstselektion von Käufern als fairer im Vergleich zur Preisdifferenzierung ohne Selbstselektion wahrgenommen, da prinzipiell alle Käufer alle möglichen Preise erhalten können (Dickson und Kalapurakal 1994). Auch aus rechtlicher Sicht ist Preisdifferenzierung mit Selbstselektion deshalb in der Regel unbedenklich.

### Einordnung dynamischer Preisgestaltung in die Formen der Preisdifferenzierung

Wie eingangs dargelegt, ist das wesentliche Unterscheidungsmerkmal dynamischer Preisgestaltung zu anderen Formen der Preisdifferenzierung, dass die Preisvariation im Vorhinein nicht angekündigt wird. So besteht der Unterschied zwischen dynamischer Preisgestaltung und der zeitlichen Preisdifferenzierung wie bspw. Peak-Load-Pricing (Skiera und Spann 1998) dahingehend, dass im Peak-Load-Pricing sich die zeitlich differenzierten Preise auf den Nutzungszeitpunkt beziehen und im Vorhinein angekündigt werden.

**Figure 2:**
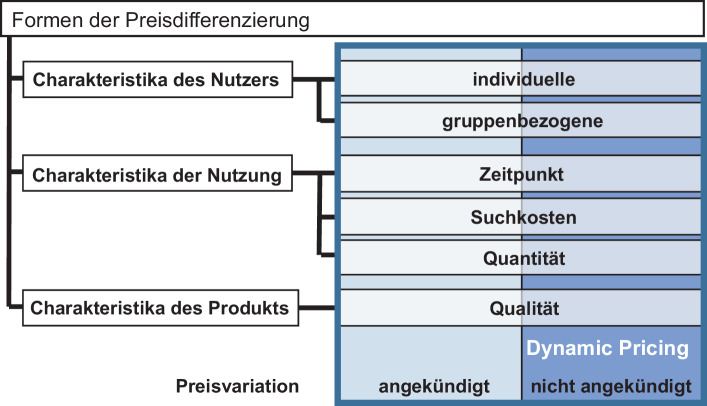

Abb. 2 stellt die unterschiedlichen Formen der Preisdifferenzierung dar. Grundsätzlich können die unterschiedlichen Dimensionen (bezogen auf Nutzer, Nutzung sowie Produkt) mit dynamischer Preisgestaltung kombiniert werden. So ist bei der Bahn ein Preisunterschied anhand der Produktqualität zwischen der 1. & 2. Klasse bekannt, die genauen Preise können sich aber je nach Kauf- und Nutzungszeitpunkt ändern (u. a. bezeichnet als „Sparpreis“ und „Supersparpreis“). Anhand von Abb. 2 wird ebenfalls deutlich, dass dynamische Preisgestaltung sowohl Formen der Preisgestaltung mit Selbstselektion (z. B. unterschiedliche Preise im Zeitverlauf) als auch ohne Selbstselektion (z. B. zu einem Kaufzeitpunkt unterschiedliche Preise für verschiedene Käufer) beinhaltet. Preis-Promotions, insbesondere wenn sie für Konsumenten zufällig erfolgen (Gedenk 2002), sind der dynamischen Preisgestaltung zuzuordnen.

## Formen der dynamischen Preisgestaltung

Wie eingangs erläutert, lässt sich dynamische Preisgestaltung anhand unterschiedlicher Preise für ein prinzipiell gleiches Produkt in Abhängigkeit beobachtbarer Umweltzustände definieren, wobei die Preise im Zeitverlauf für alle Käufer, als auch zu einem Zeitpunkt zwischen unterschiedlichen Käufern, variieren können. Diese relativ allgemeine Definition zielt auf die beobachtbare (dynamische) Varianz der Preise ab.

Zudem können zwei grundsätzliche Formen der dynamischen Preisgestaltung unterschieden werden, die zu einer dynamischen Varianz der Preise führen: (i) nicht interaktive dynamische Preise sowie (ii) interaktive dynamische Preise.

### Nicht interaktive dynamische Preise

Nicht interaktive dynamische Preise werden entweder vom Verkäufer oder einem Intermediär zwischen Verkäufern und Käufern, z. B. einer Plattform oder einem Marktplatz, festgesetzt. Käufer haben somit keinen direkten Einfluss auf die Preisgestaltung (Spann et al. 2018). In diesem Zusammenhang ist zwischen Intermediären, die als Verkäufer (d. h. An- und Verkauf auf eigene Rechnung) und Maklern (d. h. Vermittler) fungieren, zu unterscheiden. Amazon übt als Intermediär beides aus, d. h. verkauft als Händler auf eigene Rechnung sowie vermittelt zwischen Käufern und Dritthändlern als Makler. Im ersten Fall setzt der Verkäufer einen Preis, den Käufer akzeptieren (d. h. zu diesem Preis kaufen) können oder nicht. Der zweite Fall, die dynamische Preisfestsetzung durch einen Intermediär/Makler wie eine Plattform oder einen Marktplatz, beinhaltet zwei Möglichkeiten: entweder legen die Dritthändler auf der Plattform die Preise selbst fest (siehe Amazon), oder die Plattform übernimmt die Preissetzung (letzteres wird auch als „Spot-Pricing“ bezeichnet).

#### Dynamisch festgesetzte Preise

Dynamisch festgesetzte Preise entsprechen der klassischen Situation in Verkäufer-Käufer-Austauschbeziehungen, beispielsweise im Handel. Der Unterschied liegt jedoch in den häufigeren, u. U. sogar mehrmals täglichen Preisanpassungen im Vergleich zu traditionellen Situationen mit festgesetzten Preisen und einer selteneren Preisanpassung (Brynjolfsson und Smith 2000).

Die Luftverkehrsbranche hat seit mehreren Jahrzehnten dynamische Preisgestaltung im Rahmen von „Yield Management“- oder „Revenue Management“-Systemen eingesetzt. Kernstück dieser Systeme ist die Entscheidung, ob ein kapazitätsbeschränktes, häufig als verderblich bezeichnetes Produkt (z. B. Sitzplatz auf einem bestimmten Flug oder Hotelzimmer an einem bestimmten Tag) in einer günstigen Preisklasse oder nur noch in einer höheren Preisklasse angeboten werden sollte (Belobaba 1989). Aufgrund von Nachfrageprognosen und aktuellem Buchungsverhalten werden je nach Zeitpunkt der Buchung unterschiedliche Buchungsklassen angeboten, so dass sich die Preise dynamisch im Zeitverlauf ändern (Spann et al. 2005).

In den letzten Jahren hat sich die Anwendung von dynamisch festgesetzten Preisen insbesondere im Online-Handel auf Ge- und Verbrauchsgüter ausgeweitet (Chen et al. 2016): Abb. 3 stellt beispielhaft die dynamischen Preisverläufe für zwei Produkte beim Online-Händler Amazon dar.^4^ Deutlich wird, dass die Preise erheblich variieren können. So liegt der maximale Preis der mobilen Klimaanlage (Teil A) mit 457,36 € 15,8 % über dem minimalen Preis von 394,95 € und der des Wäschetrockners (Teil B) mit 467,99 € 14,4 % über dem minimalen Preis von 409 € (bei Amazon).

**Figure 3:**
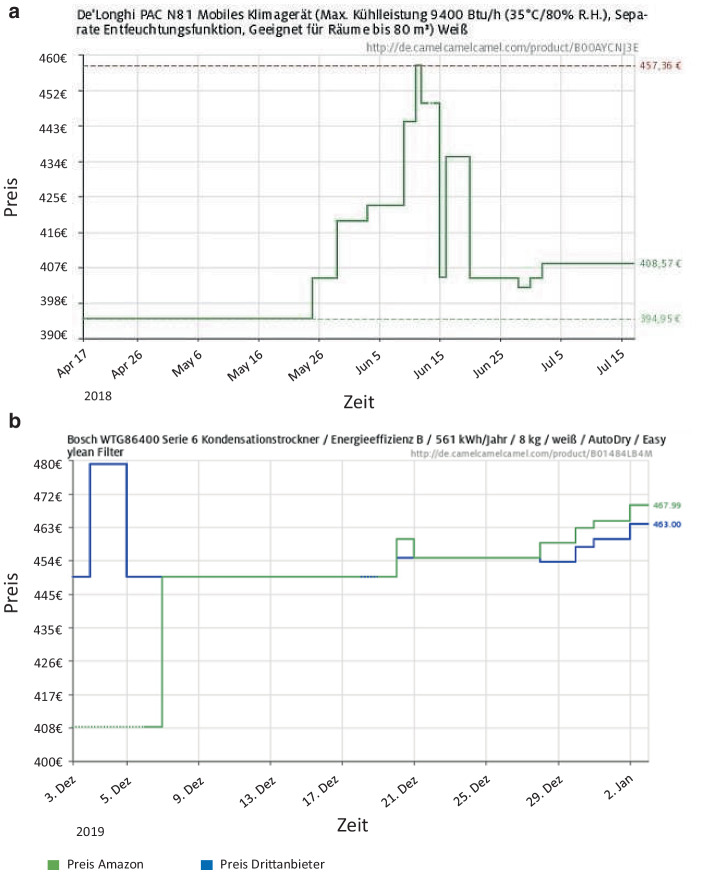

Ab Abschn. 4 wird der Fokus auf dynamisch festgesetzte Preise gelegt, da diese Form der dynamischen Preisgestaltung zurzeit die größte Beachtung von Unternehmen, Nachfragern, Verbraucherorganisation und der Politik^5^ erfährt.

#### Spot Pricing

Beim so genannten „Spot-Pricing“, auch Real-Time Pricing genannt (Schlereth et al. 2018), erfolgt die dynamische Preisfestsetzung in der Regel durch einen Intermediär, z. B. eine Plattform oder einen Marktplatz. Weder die Käufer noch die Verkäufer haben direkten Einfluss auf die Preisfestsetzung. Sie können nur dahingehend reagieren, ob sie zum aktuellen Preis kaufen (Käufer) oder ihr Produkt anbieten (Verkäufer)^6^. Die Plattform bzw. der Marktplatz verfolgen mit der dynamischen Preisgestaltung im „Spot-Pricing“ das Ziel, Angebot und Nachfrage in Einklang zu bringen (Hall et al. 2015).

Prominente Beispiele sind das „Real-Time Pricing“ im Energiebereich (Dutta und Mitra 2017) sowie vor allem „Surge-Pricing“ (UBER) bzw „Prime Time“ (Lyft) auf den Fahrdienstvermittlungsplattformen UBER und Lyft: Im Falle einer deutlichen höheren Nachfrage in einer Region erhöhen diese Fahrdienstvermittlungsplattformen regional die Preise, um dadurch eine Senkung der Nachfrage und eine Erhöhung des Angebots zu erwirken. Letzteres kann dadurch entstehen, dass zusätzliche Fahrer (d. h. Anbieter von Fahrdienstleistungen) aktiv werden oder aktive Fahrer sich in die Region des erhöhten Preises begeben und dort das Angebot erhöhen (Lu et al. 2018). Durch den Ausgleich von Angebot und Nachfrage soll die für diese Plattformen wichtige Zielgröße der durchschnittlichen Wartezeit auf ein Fahrzeug unter einem kritischen Wert von wenigen Minuten bleiben (Hall et al. 2015).

### Interaktive dynamische Preise

Im Rahmen von interaktiven dynamischen Preisen ergibt sich der Preis aufgrund der Interaktion zwischen Käufern und Verkäufern. Beispiele für interaktive Preismechanismen sind klassische Verkaufsauktionen (wie beispielsweise die Englische oder Holländische Auktion; McAfee und McMillan 1987) sowie neue im Internet entstandene Auktionsformen wie die Name-Your-Own-Price Auktion (Hinz et al. 2011) und die Generalized-Second-Price Auktion im Suchmaschinenmarketing (Edelman et al. 2007). Aufgrund des unterschiedlichen Käufer- und Verkäuferverhaltens in interaktiven Preismechanismen können sich die Transaktionspreise zwischen jeder einzelnen Auktion unterscheiden – im Fall von Name-Your-Own-Preis oder der Generalized-Second-Price Auktion findet eine Vielzahl von Auktionen in Zeitverlauf statt, so dass Preise dynamisch stark variieren können.

## Ausgestaltung dynamisch festgesetzter Preise

In diesem Kapitel wird die Ausgestaltung dynamisch festgesetzter Preise aus Verkäufersicht erörtert, da diese in jüngster Zeit stärker diskutiert wurden (siehe z. B. an der Heiden und Wersig (2018), Zander-Hayat et al. (2016)). Hierbei müssen Verkäufer verhaltenswissenschaftliche Überlegungen berücksichtigen und Entscheidungen zu den grundlegenden Preisdimensionen (inklusive der personenbezogenen dynamischen Preisdifferenzierung) sowie dem Einfluss von Kontextfaktoren treffen. Daneben sind auch mögliche Wechselwirkungen zwischen den einzelnen Faktoren zu beachten.

### Verhaltenswissenschaftliche Einflussfaktoren

Erwartungsbildung und Referenzpreiseffekte sowie Fairnesswahrnehmung sind wesentliche verhaltenswissenschaftliche Einflussfaktoren, die von Verkäufern bei dynamischer Preisanpassung zu beachten sind.

#### Erwartungsbildung und Referenzpreiseffekte

**Figure 4:**
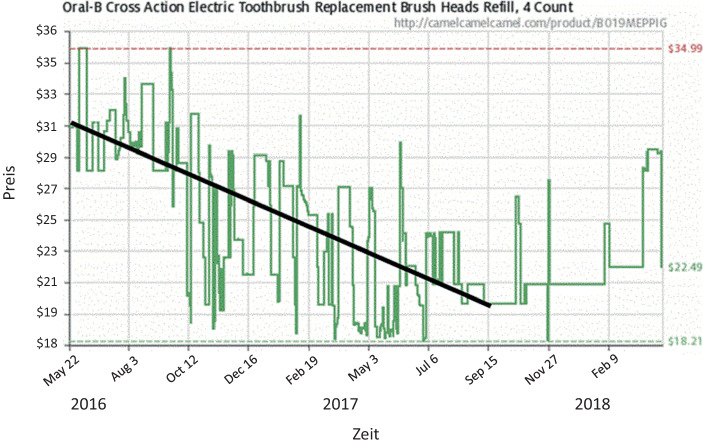

Die dynamische Preisanpassung kann die Preiserwartungen von potenziellen Käufern beeinflussen (Drechsler und Natter 2011). Hierbei ist der von Konsumenten erwartete grundsätzliche Markttrend, der von der Produktkategorie abhängt, zu berücksichtigen. Beispielsweise können bei langlebigen (technologischen) Konsumgütern fallende Preise zur Erwartung weiter fallender Preise und dadurch zu einer Kaufzurückhaltung bei potenziellen Käufern führen (siehe den fallenden Preistrend bis Mitte September in Abb. 4). Im Gegensatz dazu können bei Luftfahrtdienstleistungen zum Abflug hin steigende Preisverläufe zu einem frühzeitigen Buchungsverhalten führen (Spann et al. 2005). Volatile Preisverläufe, z. B. bei Hotelzimmern, erschweren die Erwartungsbildung.

Daneben wirkt sich die dynamische Preisgestaltung auf die internen Referenzpreise von Käufern aus, die auf Basis von in der Vergangenheit beobachteten Preisen gebildet werden (Kalyanaram und Winer 1995). Referenzpreise beeinflussen die Kaufentscheidung, da Preise von Käufern nicht nur absolut, sondern auch im Vergleich zu einem Referenzpreises beurteilt werden (Kalyanaram und Winer 1995). Darüber hinaus beeinflusst die dynamische Preisgestaltung ebenfalls externe Referenzpreise, sofern auch Wettbewerber ihre Preise dynamisch anpassen.

#### Wahrgenommene Preisfairness

Preisfairness bezeichnet das vom Käufer wahrgenommene Fairnessurteil in Bezug auf die vom Verkäufer festgesetzten Preise (Haws und Bearden 2006). Das so genannte „Dual Entitlement-Prinzip“ geht davon aus, dass Käufer Anspruch auf einen fairen Preis und Verkäufer Anspruch auf einen fairen Gewinn haben, jeweils im Vergleich zu einem Referenzpunkt (Kahneman et al. 1986). Im Rahmen der dynamischen Preisgestaltung sind die wichtigsten Referenzpunkte zur Beurteilung der wahrgenommenen Preisfairness (Haws und Bearden 2006):

- Preis(e), den/die andere Käufer gezahlt haben sowie
- Preis(e) zu anderen Zeitpunkten.

Haws und Bearden (2006) zeigen, dass (1) Preisunterschiede zwischen Käufern am unfairsten wahrgenommen werden sowie (2) eine geringe Zeitdauer zwischen Preisänderungen Fairnesswahrnehmungen verringert. Priester et al. (2020) zeigen, dass Konsumenten individuelle Preisunterschiede als weniger fair im Vergleich zu gruppenbezogenen Preisunterschieden wahrnehmen.

Kosteninduzierte Preissteigerungen werden in der Regel als fair wahrgenommen, jedoch finden Lu et al. (2019), dass bei hoher Marktmacht auch kosteninduzierte Preissteigerungen als unfair wahrgenommen werden.

Im Vergleich zu nicht interaktiven dynamischen Preisen werden interaktive dynamische Preise als fairer wahrgenommen, da die Beeinflussungsmöglichkeit durch Käufer (z. B. Gebote in Auktionen) die Preisakzeptanz erhöht (Haws und Bearden 2006).

### Grundlegende Preisdimensionen

Zur Ausgestaltung der dynamisch festgesetzten Preise muss ein Verkäufer zunächst die grundlegenden Preisdimensionen Markteinführungspreis und eine dynamische Preisstrategie wählen. Sofern der Verkäufer mehr als einen Verkaufskanal nutzt (z. B. Online- & Offline-Shop) müssen noch kanalbezogene Aspekte bei der Preisgestaltung berücksichtigt werden. Außerdem muss die Transparenz der Preisgestaltung festgelegt und über die Anwendung personenbezogener dynamischer Preisdifferenzierung entschieden werden.

#### Markteinführungspreis und dynamische Preisstrategie

Bei der Wahl des Markteinführungspreises und der langfristigen dynamischen Preisstrategie diskutiert die Preisliteratur zwei grundlegende Strategien (Simon und Fassnacht 2016): Eine Preis-Skimming-Strategie zeichnet sich durch einen hohen Markteinführungspreis aus, der sukzessive gesenkt wird. Mit dieser Preisstrategie sollen hohe Zahlungsbereitschaften zu Beginn eines Produktlebenszyklus abgeschöpft werden (Spann et al. 2015). Demgegenüber zeichnet sich eine Penetrations-Preisstrategie durch einen niedrigen Markteinführungspreis aus, um dadurch Marktanteile zu gewinnen und Skaleneffekte zu realisieren (Spann et al. 2015). Die langfristige dynamische Preisstrategie stellt den Basispreispfad dar, von dem dynamisch in Abhängigkeit von aktuellen Nachfrage‑, Angebots- und Wettbewerbsbedingungen Preise angepasst werden.

#### Kanalbezogene Preisunterschiede

Sofern ein Verkäufer mehrere Kanäle anbietet, stellt sich die Frage, inwiefern die dynamische Preisgestaltung in allen Kanälen gleichermaßen möglich und gewünscht ist. Insbesondere bei der Verwendung von Online- und Offline-Kanälen kann eine dynamische Preisgestaltung im Offline-Kanal oftmals nur eingeschränkt umgesetzt werden: Auch wenn zunehmend elektronische Preisschilder im Offline-Handel eingesetzt und damit die Kosten von Preisänderungen gesenkt werden, kann eine zu häufige Preisanpassung unerwünschte Effekte haben – beispielsweise wenn Kunden aufgrund einer dynamischen Preiserhöhung an der Kasse einen anderen Preis zahlen sollen als denjenigen, den sie zuvor gesehen hatten.

Sofern eine dynamische Preisanpassung nur im Online-Kanal umgesetzt wird, ergeben sich Preisunterschiede zum Offline-Kanal. Folglich können die Preise im Offline-Kanal das Ausmaß der dynamischen Preisanpassungsschwankungen limitieren, sofern Kunden nur eine maximale Preisdifferenz zwischen Online- und Offline-Kanal akzeptieren (Homburg et al. 2019). Zusätzlich kann der Verkäufer versuchen, durch unterschiedliche Produktnummern und Eigenmarken die Preistransparenz zwischen beiden Kanälen zu reduzieren. So zeigen beispielsweise Ringel und Skiera (2016), dass auf Preisvergleichsseiten mittlerweile rund 1500 verschiedene Waschmaschinen oder Staubsauger, darunter viele Eigenmarken, angeboten werden.

#### Transparenz der Preisgestaltung

Eine wichtige Entscheidung aus Verkäufersicht im Rahmen der dynamischen Preisgestaltung ist, ob die Anwendung dynamischer Preise gegenüber den Käufern offengelegt werden soll oder nicht. Aufgrund möglicher negativer Auswirkungen auf die wahrgenommene Preisfairness (siehe Abschn. 4.1.2) entscheidet sich die Mehrzahl der Verkäufer gegen eine explizite Offenlegung der Anwendung der dynamischen Preisgestaltung.

Diese geübte Praxis der Nichtoffenlegung kann allerdings kritisch hinterfragt werden. Hinz et al. (2011) zeigen in einem Labor- und einem Feldexperiment, dass die Offenlegung der dynamischen Preisgestaltung – in ihrem Anwendungsfall einer Name-Your-Own-Price Auktion^7^ – zu sowohl höherem Gewinn des Verkäufers als auch einer höheren Kundenzufriedenheit führen kann. Hinz et al. (2011) erklären dieses Ergebnis dadurch, dass im Falle der Offenlegung Bieter informierter Entscheidungen treffen, da sie wissen, in welchem „Spiel“ sie sich befinden. Darüber hinaus führt die dynamische Preisgestaltung dazu, dass mehr Bieter erfolgreich sind und somit die Markteffizienz höher ist als im Vergleich zur Nichtoffenlegung sowie einer statischen Preisgestaltung. Zander-Hayat et al. (2016) argumentieren außerdem, dass aus rechtlicher Sicht Konsumenten über eine personalisierte Preissetzung informiert werden müssen.

Folglich kann die Offenlegung der dynamischen Preisgestaltung eine sinnvolle Strategie für Verkäufer darstellen und möglichen regulatorischen Anforderungen zur Offenlegung zuvorkommen.

#### Dynamische personenbezogene Differenzierung der Preise

Verkäufer müssen entscheiden, ob eine personenbezogene Differenzierung der Preise erfolgen soll (d. h. verschiedene Käufer zahlen zu einem Kaufzeitpunkt unterschiedliche Preise) oder nicht (d. h. zu einem Kaufzeitpunkt zahlen alle Käufer den gleichen Preis). Die wesentlichen Kriterien für diese Entscheidung sind Abschöpfung der Zahlungsbereitschaftsunterschiede zwischen Käufern, Datenanforderungen, rechtlicher Überlegungen und Konsumentenakzeptanz.

Der *Verzicht auf eine personenbezogene Differenzierung* hat den Nachteil, dass Unterschiede in Zahlungsbereitschaften zwischen Käufern nicht ausgenutzt werden können und somit die theoretisch mit der personenbezogenen Differenzierung verbundenen Steigerungen der Gewinne nicht möglich sind (cf. Pigou 1929). Empirisch ist es natürlich offen, ob diese theoretisch möglichen Gewinnsteigerungen überhaupt realisiert werden können, da dafür die Zahlungsbereitschaften auch bekannt sein müssen, was eine hinreichend gute Messung erfordert.

Dynamische Preisgestaltung ohne personenbezogene Differenzierung ist unkritischer in der Datennutzung, da lediglich aggregierte Nachfrageprognosen bzw. Schätzungen der Preissensibilität erforderlich sind. Damit ist diese Praxis auch gleichermaßen anwendbar für anonyme (Neu‑)Kunden sowie bestehende und damit bekannte Kunden.

Zudem sollte bei einer nicht-personenbezogenen dynamischen Preisgestaltung die wahrgenommene Preisfairness höher liegen, da, wie in Abschn. 4.1.2 erläutert, Preisunterschiede zwischen Käufern am unfairsten wahrgenommen werden (Haws und Bearden 2006). Dies sollte auch positiv auf die Kundenakzeptanz der dynamischen Preisgestaltung wirken.

Die Motivation für eine *personenbezogene Differenzierung* der Preise besteht in der Gewinnsteigerung, die durch eine Ausnutzung von Unterschieden in den Zahlungsbereitschaften zwischen Käufern ermöglicht wird (cf. Pigou 1929).

Allerdings hat diese Vorgehensweise mehrere Nachteile. Erstens können Zahlungsbereitschaften normalerweise nicht direkt beobachtet werden.^8^ Daher muss auf andere Variablen als Indikator für die Zahlungsbereitschaft zurückgegriffen werden. Im Online-Handel stehen beispielsweise das Betriebssystem bzw. Zugriffsgerät (z. B. iOS vs. Android; Desktop, Mobile, Tablet), demographische Informationen, Standort des Nutzers, sowie vergangenes Suchverhalten (als Indikator von Produktinteresse) oder Kaufverhalten (u. a. Neukunde vs. Bestandkunde) zur Verfügung. Allerdings ist unklar, wie gut diese Indikatoren geeignet sind, Zahlungsbereitschaften zu identifizieren.

Zweitens ist eine personenbezogene dynamische Preisdifferenzierung aus rechtlicher Sicht kritisch zu betrachten. Die Verwendung von demographischen Indikatoren (z. B. Alter und Geschlecht) verstößt mit hoher Wahrscheinlichkeit gegen das Diskriminierungsverbot. Darüber hinaus erfordert eine personenbezogene dynamische Preisdifferenzierung die Verwendung personenbezogener Merkmale und somit entsprechende Einwilligungen der (potenziellen) Käufer im Rahmen der Datenschutz-Grundverordnung (DSGVO). Die Bereitschaft dazu wird niedrig sein, da die potenziellen Käufer ja damit rechnen müssen, deswegen ggf. höhere Preise zu bezahlen.

Drittens besteht die Gefahr, dass Kunden eine entsprechende personenbezogene dynamische Preisdifferenzierung aufdecken, wenn beispielsweise anhand des Betriebssystems differenziert wird und ein Käufer ein iOS Firmen-Smartphone und ein privates Android-Gerät besitzt. Die Kundenreaktion in Folge einer solchen Aufdeckung ist vermutlich stark negativ und kann sich über soziale Medien schnell verbreiten, so dass der Reputationsschaden für das Unternehmen erheblich sein kann (Martin et al. 2018). Insofern bieten sich nur sozial akzeptierte Kriterien für niedrigere Preise, z. B. die schon weiter oben erwähnten Preisnachlässe für Familien, Studierende und sozial Benachteiligte, an.

In Anbetracht dieser Nachteile ist es nicht überraschend, dass Zander-Hayat et al. (2016) auch feststellen, dass es nur vereinzelt Nachweise über personalisierte Preise gibt. Eine Alternative zur personenbezogenen Preisdifferenzierung ist allerdings das personenbezogene Gewähren von Rabatten, beispielsweise über individualisierte Coupons. In einem solchen Fall wäre der allen Konsumenten angebotene Preis gleich, aber die Höhe der individualisierten Coupons unterschiedlich und so letztlich auch der effektiv zu zahlende Preis. Eine solche Vorgehensweise vermeidet eine ganze Reihe der oben aufgeführten Nachteile.

### Kontextfaktoren für die dynamische Preisgestaltung

Kontextfaktoren für die dynamische Preisgestaltung sind mögliche Reaktionen auf Nachfrageänderungen, Änderung der Angebotsbedingungen, Produktcharakteristika und des Wettbewerbsverhaltens.

#### Nachfrage

Änderungen in der Preissensibilität und Nachfrageschocks (also Nachfrageveränderungen) sind wesentliche ökonomische Einflussfaktoren auf dynamische Preisanpassungen. Änderungen der Preissensibilität können dabei von Umwelt- bzw. Kontextfaktoren ausgelöst werden. Teil A von Abb. 3 stellt den Preisverlauf für eine Klimaanlage dar. Deren Preisanstieg ab Ende Mai könnte durch eine höhere Nachfrage aufgrund sommerlicher Temperaturen ausgelöst worden sein. Sofern eine personenbezogene dynamische Preisanpassung auf Basis von Kundencharakteristika erfolgen soll, werden Preise auf Basis nachfragebezogener Indikatoren der Preissensibilitäten, beispielsweise dem vergangenen Kauf- und Bestellverhalten, angepasst.

#### Angebot

Angebotsbezogene Einflussfaktoren auf die dynamische Preisgestaltung sind Lagerbestandsänderungen, das Ende der Verkaufsperiode sowie Kostenänderungen bei Einkauf bzw. Herstellung. Beispielsweise kann die Preissenkung der Klimaanlage ab Mitte/Ende Juni in Teil A von Abb. 3 durch kälteres Wetter (nachfragebezogene Preisanpassung), durch die Erhöhung des Lagerbestands (Angebot: Eintreffen einer neuen Lieferung vom Hersteller) oder in Reaktion auf Preisveränderungen des Wettbewerbs verursacht worden sein.

#### Produkt

Produktcharakteristika, die einen Einfluss auf die dynamische Preisgestaltung ausüben sind insbesondere die Produkthaltbarkeit bei verderblichen Produkten sowie technische Neuerungen. Darüber hinaus ist denkbar, dass Produktcharakteristika über die Zeit unterschiedlich wichtig sind. So weisen beispielsweise Nicht-Diesel-Autos besonders hohe Vorteile gegenüber Diesel-Autos auf, wenn Fahrverbote für Diesel-Autos vorliegen. Gleiches gilt für besonders gute FFP („Filtering Face Piece“)-Schutzklassen für Atemmasken in Zeiten von Pandemien wie COVID-19.

#### Wettbewerb

Insbesondere auf Märkten bei denen vergleichbare oder gleiche Produkte von mehreren Verkäufern angeboten werden, stellen die Preise der Wettbewerber einen bedeutenden Einflussfaktor auf dynamische Preisanpassungen dar (Chen et al. 2016).

Teil B von Abb. 3 stellt den Preisverlauf für einen Wäschetrockner dar. Ab Ende Dezember ist ein Zusammenhang der Preise zwischen dem Drittanbieter und Amazon ersichtlich: Amazon scheint einem Preisanstieg des Drittanbieters zu folgen (oder umgekehrt), wobei der Preis von Amazon jeweils wenige Euro über dem Preis des Drittanbieters liegt.^9^

Die Bedeutung und Stärke von Wettbewerbspreisen auf das Ausmaß der dynamischen Preisanpassung hängt von der Preistransparenz im Markt ab. Märkte mit hoher Preistransparenz (wie beispielsweise auf dem Marktplatz von Amazon) zeigen hier stärkere wettbewerberbedingte Preisänderungen (siehe Teil B von Abb. 3 sowie Chen et al. 2016) als Märkte mit geringerer Preistransparenz (beispielsweise unverpackte Lebensmittel wie Obst und Gemüse).

## Beurteilung dynamischer Preisgestaltung

Nachfolgend erfolgt eine Beurteilung der dynamischen Preisgestaltung aus Verkäufer- und Käufersicht mit einem Fokus auf dynamisch festgesetzte Preise (siehe Tab. 1). Zur Beurteilung der dynamischen Preisgestaltung aus Verkäufersicht werden die Kriterien Gewinnsteigerungspotenzial, (technische) Umsetzbarkeit, Daten und rechtliche Anforderungen, Käuferakzeptanz und Wettbewerbsimplikationen herangezogen. Die Kriterien aus Käufersicht sind Marktdurchdringung, Anforderungen an die Käufer sowie Datenschutzaspekte.

**Table 1:** 

	*Kriterium*	*Beurteilung dynamischer Preisgestaltung*	
		Nicht personenbezogen	Personenbezogen
Verkäufersicht	Gewinnsteigerungspotenzial	Hohes Gewinnsteigerungspotenzial	Höheres Gewinnsteigerungspotenzial
	Technische Umsetzbarkeit	Gute Umsetzbarkeit durch vorhandene Softwaresysteme (u. a. Drittanbieter)	Leichter umsetzbar über individualisierte Rabatte
	Daten	Gute Datenverfügbarkeit online	Verfügbare Indikatoren wie vergangenes Such- und Kaufverhalten, Standort & Kontext gestatten häufig keine besonders gute Prognose der Zahlungsbereitschaft
	Rechtliche Anforderungen, insbesondere aufgrund von DSGVO	Unkritisch	Einwilligung des Konsumenten zur Verarbeitung der Daten notwendig
	Akzeptanz	Offenlegung der dynamischen Preisgestaltung kann Akzeptanz erhöhen	Akzeptanz kritisch; leichter umsetzbar über individuelle Rabatte
	Wettbewerbsimplikationen	Erhöht Preiswettbewerb, insb. in Situationen mit hoher Preistransparenz	Wettbewerbsimplikation unklar, da Preistransparenz häufig gering ist
Käufersicht	Marktdurchdringung	Hohe Marktdurchdringung (weniger Käufer werden durch Preis ausgeschlossen)	
	Anforderungen	Stärkere Erfordernis des Preisvergleichs (aber bessere Vergleichsmöglichkeit in digitalen Medien)	
	Privacy	Unkritisch	Preisgabe von personenbezogenen Daten (mit Einwilligung)

### Beurteilung aus Verkäufersicht

Wesentliche Kriterien zur Beurteilung der dynamischen Preisgestaltung aus Verkäufersicht sind Gewinnsteigerungspotenzial, (technische) Umsetzbarkeit, Daten und rechtliche Anforderungen, Käuferakzeptanz und Wettbewerbsimplikationen.

Die Anwendung dynamischer Preisgestaltung erhöht den Gewinn des Anbieters, da durch stärker differenzierte Preise – im Vergleich zu keiner oder rein statischer Preisdifferenzierung – zumindest theoretisch besser Zahlungsbereitschaften abgeschöpft werden können (cf. Pigou 1929). Im Vergleich zur nicht-personenbezogenen Differenzierung ist die Gewinnsteigerung bei der personenbezogenen dynamischen Preisdifferenzierung noch höher.

Die technische Umsetzbarkeit dynamischer Preisgestaltung ist auch für kleinere Händler aufgrund der Verfügbarkeit von Drittanbieter-Softwaresystemen gegeben^10^. Eine personenbezogene Preisdifferenzierung ist über individualisierte Rabatte umsetzbar, da diese die Transparenz individuell differenzierter Preise verringern und somit auch interpersonelle Vergleiche (mit entsprechenden negativen Auswirkungen auf die wahrgenommene Preisfairness) erschweren.

Im Online-Handel ist aufgrund der digitalen Messbarkeit des Kundenverhaltens die Datenverfügbarkeit als gut für die Anwendung dynamischer Preisgestaltung einzuschätzen. Für eine personenbezogene dynamische Preisdifferenzierung können vergangenes Such- und Kaufverhalten sowie der aktuelle Standort und Kontext (z. B. Wetter oder sozialer Kontext) als Indikatoren der Zahlungsbereitschaft dienen (Zubcsek et al. 2017). So könnten beispielsweise Kaufabbrüche nach dem Füllen des Warenkorbs als Indikator dafür angesehen werden, dass der Gesamtpreis, bestehen aus den Preisen für die Produkte und den Versand, zu hoch war. Das Versenden von Coupons im Anschluss an solche Kaufabbrüche könnte dann doch noch den gewünschten Verkauf herbeiführen.

Dynamische Preisgestaltung ohne personenbezogene Differenzierung erfordert auch keine personenbezogenen Daten für die Preisoptimierung und ist daher im Hinblick auf die Datenschutz-Grundverordnung (DSGVO) unkritisch. Eine personenbezogene dynamische Preisdifferenzierung erfordert die Verwendung personenbezogener Daten und folglich Einwilligungen der (potenziellen) Käufer in die Nutzung deren Daten gemäß Datenschutz-Grundverordnung (DSGVO).

Die Akzeptanz dynamischer Preisgestaltung ist höher bei nicht personenbezogener dynamischer Preisgestaltung im Vergleich zur personenbezogenen dynamischen Preisgestaltung. Eine Offenlegung der nicht personenbezogenen dynamischen Preisgestaltung kann auch positiv auf die Akzeptanz wirken. Eine personenbezogene dynamische Preisgestaltung sollte über individuelle Rabatte erfolgen. Mögliche Effekte der dynamischen Preisgestaltung auf Erwartungsbildung und Referenzpreise sind zu berücksichtigen (siehe auch Forschungsbedarf in Tab. 2 unten).

**Table 2:** 

*Bereich*	*Themen*
Algorithmen und KI	Algorithmische Aversion
	Algorithmische Kollusion
Daten & Privacy	Privacy-schützende dynamische Preisgestaltung
	Kosten-Nutzen-Analyse
Entscheidungsverhalten & -verzerrungen	Effekt auf Erwartungsbildung und Referenzpreise
	Identifikation von Entscheidungsverzerrungen
	De-Biasing (z. B. bei der Erwartungsbildung)
Anwendung	Zusammenwirken dynamischer Preise in Offline- und Online-Kanal
	Ermittlung weiterer Anwendungsgebiete für die dynamische Preisgestaltung

Die dynamische Preisgestaltung erhöht den Preiswettbewerb insbesondere bei hoher Markttransparenz wie beispielsweise auf dem Amazon-Marktplatz. Bei geringer Markttransparenz sind die Wettbewerbsimplikationen unklar. Individualisierte Rabatte können die Markttransparenz reduzieren, da die Höhe dieser Rabatte für den Wettbewerb normalerweise nicht beobachtbar ist. Das könnte starke Wettbewerberreaktionen verringern. Weitere mögliche Implikationen der dynamischen Preisgestaltung können Effekte auf die Platzierung (Anzeigenrang) auf einem Marktplatz oder in einer Preissuchmaschine sein (Chen et al. 2016)^11^.

### Beurteilung aus Käufersicht

Aus Käufersicht sind Marktdurchdringung, Anforderungen an die Käufer sowie Datenschutzaspekte zu diskutieren.

Die dynamische Preisgestaltung kann für Käufer von Vorteil sein, die bei nicht dynamischen Preise durch einen Preis über ihrer Zahlungsbereitschaft vom Kauf ausgeschlossen wären (siehe auch Abschn. 2.1). So ist beispielsweise nicht klar, was passieren würde, wenn die Deutsche Bahn nicht mehr in die beiden Produktvarianten erste und zweite Klasse unterscheiden dürfte. Denkbar wäre beispielsweise, dass der dann gesetzte einheitliche Preis für eine Bahnfahrt zwischen den jetzigen Preisen der ersten und zweiten Klasse liegt. Konsumenten mit einer Zahlungsbereitschaft in Höhe der jetzigen Preise der zweiten Klasse könnten dann nicht mehr Bahnfahren. Somit kann dynamische Preisgestaltung zu einem breiteren Zugang zu Produkten und somit einer größeren Marktdurchdringung führen, die insbesondere Konsumenten mit niedrigeren Zahlungsbereitschaften hilft.

Für Käufer steigen allerdings die Anforderungen, da eine dynamische Preisgestaltung zu stärker schwankenden Preisen führt. Das Realisieren niedriger Preise erfordert eine intensivere Suche, die aber auch durch Preisvergleichsseiten oder Angebote wie Preisalarme, die beispielsweise eine E‑Mail auslösen, wenn Preise unter bestimmte vom Konsumenten gesetzte Preisschwellen gefallen sind, erleichtert wird.

Dynamische Preisgestaltung ohne personenbezogene Differenzierung ist aus Sicht des Datenschutzes („Privacy“) unkritisch, da keine personenbezogenen Daten erforderlich sind. Demgegenüber ist eine personenbezogene dynamische Preisdifferenzierung mit der Verwendung persönlicher Daten verbunden und erfordert aufgrund der seit 2018 geltenden Datenschutz-Grundverordnung (DSGVO) die Einwilligungen des Nutzers.

## Zusammenfassung und Forschungsbedarf

Dieser Beitrag stellt die ökonomischen Grundlagen der dynamischen Preisgestaltung dar, klassifiziert Ausgestaltungsmöglichkeiten, diskutiert ökonomische und verhaltenswissenschaftliche Einflussfaktoren sowie rechtliche und datenbezogene Aspekte. Darüber hinaus erfolgt eine Beurteilung der dynamischen Preisgestaltung aus Verkäufer- und Käufersicht.

Es bleibt festzuhalten, dass neben ökonomischen insbesondere auch verhaltenswissenschaftliche Aspekte bei der Anwendung der dynamischen Preisgestaltung zu beachten sind. Dynamische Preise beeinflussen über die Erwartungsbildung und Referenzpreise das Verhalten potenzieller Käufer. Daneben hängt die Akzeptanz der dynamischen Preisgestaltung insbesondere von der wahrgenommenen Preisfairness ab. Ein bislang unterschätzter Aspekt sind mögliche positive Auswirkungen einer Offenlegung der Praxis der dynamischen Preisgestaltung. Bei der Unterscheidung zwischen personenbezogener und nicht personenbezogener dynamischer Preisdifferenzierung ist eine nicht personenbezogene dynamische Preisdifferenzierung aus Akzeptanz- und Daten(schutz)gesichtspunkten vorzuziehen, wohingegen eine personenbezogene dynamische Preisdifferenzierung über individuelle Rabatte, beispielsweise über individualisierte Coupons, umgesetzt werden kann.

Grundsätzlich kann eine dynamische Preisgestaltung in einem digitalen Umfeld aufgrund der guten Datenverfügbarkeit gut umgesetzt werden. Die systematische Anwendung von Feldexperimenten (A/B-Testing) sowie der Einsatz „lernender“ Algorithmen begünstigen dabei die optimale Ausgestaltung entsprechender Ansätze. Daneben werden Konsumenten zunehmend Erfahrung mit dynamischer Preisgestaltung machen, was sich positiv auf die grundsätzliche Akzeptanz auswirken sollte. Folglich gehen wir davon aus, dass dynamische Preisgestaltung überall dort eingesetzt werden kann und wird, wo Preisdifferenzierung technisch möglich und rechtlich zulässig ist.

Allerdings besteht noch erheblicher Forschungsbedarf im Zuge dieses noch relativ neuen preispolitischen Instruments. Tab. 2 stellt den Forschungsbedarf zur dynamischen Preisgestaltung dar.

Einen ersten vielversprechenden Forschungsbereich stellen Algorithmen und künstliche Intelligenz dar. Beispielhaft sind Fragen zur Wahrnehmung und Ausgestaltung von dynamischen Preisgestaltungsalgorithmen um algorithmische Aversion zu vermeiden (Dietvorst et al. 2018) zu nennen. Algorithmische Aversion bezeichnet das Phänomen, dass Konsumenten die Interaktion mit Algorithmen ablehnen, selbst wenn Konsumenten hieraus keine Nachteile oder sogar Vorteile entstehen können. Aus regulatorischer Sicht ist hoch relevant, inwiefern das Aufeinandertreffen selbstlernender dynamischer Preisalgorithmen zu algorithmischer Kollusion bspw. in Form von überhöhten Preisen (Calvano et al. 2018; Miklós-Thal und Tucker 2019) führen und wie eine solche algorithmische Kollusion vermieden werden kann.

Im Bereich Datenschutz ist die Entwicklung von Privatsphäre-schützenden dynamischen Preisindividualisierungsanwendungen ein relevanter Bereich. Beispielsweise kann das dahingehend umgesetzt werden, dass die privaten Daten nur Nutzerseitig in einer App vorrätig gehalten werden und in der App auch die Preisindividualisierung erfolgt (vgl. vergleichbaren Ansatz bei Sutanto et al. (2013)). Ebenso ist eine Kosten-Nutzen-Analyse, d. h. Vergleich der Konsumentenrente bei dynamischer Preisgestaltung im Verhältnis zu den wahrgenommenen Kosten der Datenoffenlegung, wissenschaftlich interessant (vgl. vergleichbaren Ansatz bei Brynjolfsson et al. (2019)).

Ein weiterer Bereich von großem wissenschaftlichem Interesse ist die Analyse der Effekte dynamischer Preisgestaltung auf die Erwartungsbildung und die Referenzpreise. Außerdem können Verzerrungen im Entscheidungsverhalten von Konsumenten („behavioral biases“) wie beispielsweise so genannte „Belief-based Biases“ (d. h. Abweichungen vom rationalen Entscheidungsverhalten aufgrund verzerrter Erwartungen) im Hinblick auf die Preiserwartungen starke Auswirkungen auf die Effizienz von dynamischen Preisgestaltungsmechanismen haben (Dowling et al. 2020). Damit verbunden ist die Analyse möglicher Ansätze zum „De-Biasing“, d. h. von Gestaltungsmaßnahmen, die Entscheidungsverzerrungen von Konsumenten abschwächen.

Forschungsbedarf besteht auch in der Untersuchung der Zusammenwirkung dynamischer Preise in Offline- und Online-Kanälen. Wie kann dynamische Preisgestaltung im Offline-Handel (z. B. über dynamische Preisschilder oder Preise nur in einer App) umgesetzt werden und was sind mögliche Multi-Kanal-Aspekte bei gleichzeitiger dynamischer Preisgestaltung im Online-Kanal? Außerdem kann dynamische Preisgestaltung in unterschiedlichen Anwendungsbereichen (z. B. Entertainmentbranche) untersucht werden.

## Footnotes

1 Vergleichbare Definitionen von „dynamischer Preisgestaltung“ geben beispielsweise Haws und Bearden (2006), S. 305 („Dynamic pricing is defined here as a strategy in which prices vary over time, consumers, and/or circumstances“), oder Kannan und Kopalle (2001), S. 63 („… dynamic pricing is defined as a pricing strategy in which prices change either over time, across consumers, or across product/service bundles“).

2 Siehe bspw. https://www.br.de/radio/bayern2/sendungen/notizbuch/dynamic-pricing-wie-der-verbraucher-online-verwirrt-wird-100.html sowie https://www.marktwaechter.de/sites/default/files/marktwaechter-untersuchung-dynamische-preisdifferenzierung.pdf.

3 Entsprechend der Annahmen des mikroökonomischen Standardmodells kaufen nutzenmaximierende Konsumenten nur, falls eine nicht-negative Konsumentenrente realisiert werden kann (so genannten „Individual Rationality Constraint (IR)“) und wählen bei mehreren Alternativen, die diese Bedingung erfüllen, diejenige aus, welche die Konsumentenrente maximiert („Individual Compatibility Constraint (IC)“; vgl. Skiera (1999), S. 41 ff.).

4 Preise von Online-Händlern und auf Onlineplattformen (insbesondere Amazon) werden u. a. von CamelCamelCamel (https://de.camelcamelcamel.com) und Keepa (https://keepa.com) systematisch erfasst.

5 Siehe bspw. Ausschreibung eines Forschungsvorhabens zum Thema „Empirie zu personalisierten Preisen im E‑Commerce“ des Bundesamts für Justiz (http://www.evergabe-online.de/tenderdetails.html?id=236280).

6 Hierbei sei zu erwähnen, dass beispielsweise der Händlermarktplatz von Amazon *kein* Beispiel für dynamische Preisgestaltung mittels „Spot-Pricing“ ist, weil auf diesem Marktplatz die Verkäufer ihre Preise selbst bestimmen können.

7 Im Rahmen einer Name-Your-Own-Price Auktion muss ein Käufer einen geheimen Mindestpreis des Verkäufers überbieten, damit das Gebot erfolgreich ist. In der Studie von Hinz et al. (2011) wurde in zwei experimentellen Gruppen dieser geheime Mindestpreis an die Käufer auf Basis ihres Gebotsverhaltens individuell und damit dynamisch angepasst, wobei in einer Gruppe diese Praxis offengelegt wurde, jedoch nicht in der anderen Gruppe. Zwei weitere experimentelle Gruppen verwendeten einen einheitlichen und damit statischen Mindestpreis.

8 Eine Ausnahme stellen sogenannten „truth-telling“-Mechanismen (auch anreizkompatible Mechanismen genannt) wie beispielsweise die Vickrey-Auktion dar, die aber auch von Käufern verstanden werden müssen (Skiera und Revenstorff 1999).

9 Der positive Preisunterschied lässt sich dadurch erklären, dass Amazon trotz eines höheren Preises aufgrund der stärkeren Marke, z. B. aufgrund eines höheren Vertrauens der Konsumenten in die Abwicklung oder einer schnelleren Liefergeschwindigkeit, ausreichend Käufer gewinnen kann (Brynjolfsson und Smith 2000).

10 Beispielsweise findet sich u. a. eine Übersicht über Preisoptimierungssoftware für dynamische Preisgestaltung auf https://www.capterra.com/pricing-optimization-software/.

11 Der in diesem Zusammenhang auch zu findende Begriff des „Repricing“ bezeichnet in der Regel Preisalgorithmen, die auf wenige oder nur eine Zielvariable wie bspw. den Anzeigenrang eines Anbieters in einer Preissuchmaschine optimieren.
